# Associations Between Parental and Child Food Neophobia and Children’s Eating Behavior and Psychological Symptoms: A Cross-Sectional Mediation Analysis of South Korean Parent–Child Dyads

**DOI:** 10.3390/nu18213494

**Published:** 2026-10-24

**Authors:** Mi-Sun Lee, Kazimierz Wilkoszewski, Natalia Ziółkowska, Soo-Young Bhang, Kamilla Bargiel-Matusiewicz

**Affiliations:** 1 Faculty of Psychology, University of Warsaw, 02-097 Warsaw, Poland; dlaltjs1010@gmail.com (M.-S.L.); k.wilkoszews@student.uw.edu.pl (K.W.); 2 Addiction Policy Lab, Department of Psychiatry, College of Medicine, The Catholic University of Korea, Seoul 06591, Republic of Korea; 3 Faculty of Psychology, SWPS University, 03-815 Warsaw, Poland; nziolkowska@swps.edu.pl; 4 Department of Psychiatry, Nowon Eulji University Hospital, School of Medicine, Eulji University, Seoul 01830, Republic of Korea; 5 Eulji Psychiatry and Medical Science Center, Eulji University, Seongnam 13135, Republic of Korea

**Keywords:** food neophobia, child eating behavior, psychological symptoms, cross-sectional study, mediation analysis, parent–child dyads

## Abstract

**Objectives**: Pediatric eating behavior and psychological adjustment may be related to family food-related attitudes. This cross-sectional study examined associations between parental and child food neophobia (FN) in South Korean parent–child dyads and evaluated whether child FN statistically mediated associations between parental FN and child eating-behavior and psychological-symptom indicators. **Methods**: A cross-sectional study was conducted on a sample of 300 parent–child dyads aged 6–12 years recruited through community mental health and counseling centers. Parent-reported FN, CEBQ indicators, and PSC-17 symptoms were analyzed using multivariable linear regression and 5000-resample bias-corrected bootstrap mediation models. **Results**: Parental FN was positively associated with parent-reported child FN (B = 0.31, 95% CI 0.21–0.42). In the mutually adjusted PSC-17 model, parental FN (B = 1.09, 95% CI 0.26–1.93) and child FN (B = 0.80, 95% CI −0.02–1.62) showed positive associations with PSC-17 Total; the child-FN association was not conventionally statistically significant (*p* = 0.056). The bootstrap indirect association through child FN was B = 0.212 (95% bias-corrected bootstrap CI 0.015–0.468). For the exploratory CEBQ Total, the corresponding indirect association was B = −0.706 (95% bias-corrected bootstrap CI −1.142 to −0.334). **Conclusions**: The findings indicate cross-sectional associations and statistically detectable indirect associations, but they do not establish temporal transmission or causality. Longitudinal and intervention studies are needed to determine whether these patterns are reproducible and temporally ordered.

## 1. Introduction

Establishing healthy eating patterns during childhood is fundamental to shaping long-term physical growth, cognitive development, and emotional well-being [1]. However, a considerable number of children remain reluctant to consume unfamiliar foods or refuse to do so, a behavioral disposition referred to as food neophobia [2,3]. This avoidance of novel foods has been linked to reduced dietary variety, inadequate nutrient intake, and conflictual mealtime dynamics that can persist into adolescence and adulthood [4]. Previous research has identified multiple contributors to food neophobia, including temperamental traits (e.g., low openness to experience, anxiety and neuroticism), sensory sensitivity, parental feeding practices, presence of food allergies, as well as biological and cultural influences [5,6,7,8]. Genetic heritability of food neophobia is estimated to be around 71% to 78% [9,10]. Importantly, converging evidence suggests that parents’ own food-related attitudes and behaviors substantially influence children’s acceptance of new foods through observational learning, emotional modeling, and shared mealtime experiences [11,12].

In South Korea, rapid socioeconomic and dietary changes have occurred alongside continuing concerns about children’s eating patterns and psychological well-being [13,14]. Korean studies have reported food selectivity and picky-eating behaviors in children, while national surveillance has documented substantial mental-health concerns among young people [13,14]. Related work in East Asian behavioral research also highlights the potential relevance of family expectations and broader cultural context when interpreting child and family behaviors [15]. However, these sources do not establish that psychological symptoms in Korean children are specifically attributable to eating-related distress. Food neophobia should also be distinguished from picky/selective eating and from avoidant/restrictive food intake disorder (ARFID): food neophobia refers specifically to reluctance to try unfamiliar foods, whereas picky/selective eating is broader and ARFID is a clinically defined feeding/eating disorder. Accordingly, the extent to which parental food-related attitudes are associated with children’s food neophobia and emotional or behavioral symptoms remains insufficiently characterized in Korean samples.

Despite these concerns, relatively few empirical studies in Asia—particularly in South Korea—have examined parent–child food-neophobia associations together with child eating and psychological indicators [16,17]. International research suggests that food neophobia may co-occur with restricted dietary variety and with aspects of anxiety, negative affect, and psychological adjustment [2,18,19]. However, cross-cultural and parent–child evidence remains limited, and many instruments and studies have originated in European settings [3]. Sociocultural factors such as collective eating norms, family food practices, and parenting expectations may shape parent–child food-related behaviors, but these contextual mechanisms were not directly measured in the present study [15,20]. These gaps support the need for country-specific observational research examining the statistical associations among parental FN, child FN, eating behavior, and psychological symptoms.

Accordingly, the present cross-sectional study aimed to examine associations between parental and child food neophobia and to evaluate whether child food neophobia statistically mediated associations between parental food neophobia and child eating-behavior and psychological-symptom indicators in South Korean parent–child dyads. We specified two associational hypotheses. First, higher parental food neophobia would be associated with higher child food neophobia and with CEBQ-based eating-behavior indicators. Second, higher parental food neophobia would be associated with pediatric psychological-symptom scores, with child food neophobia examined as a potential statistical mediator. These hypotheses describe patterns of cross-sectional association rather than temporal or causal transmission.

## 2. Materials and Methods

### 2.1. Recruitment and Sampling Procedures

This cross-sectional study was conducted in South Korea between April and December 2025. Participants were recruited through community mental health and counseling centers rather than through probability sampling of the general population. The available study documentation does not establish population-based geographic coverage or the specific reasons for each family’s contact with the participating centers; these factors were therefore treated as potential sources of selection bias. Eligible participants were parents or primary caregivers aged ≥18 years who were responsible for household food purchasing and children aged 6–12 years without diagnosed chronic medical or psychiatric conditions requiring ongoing treatment. Exclusion criteria included intellectual disability or developmental disorders affecting questionnaire completion and parental refusal of written informed consent. A total of 350 families were approached, 305 consented, and 300 complete parent–child dyads were retained for analysis. No formal a priori power calculation was documented; therefore, the sample size was determined by the available completed dyads rather than by a prespecified power target. The primary analysis examined the parent–child FN association, whereas mediation analyses were considered exploratory. The final sample should not be interpreted as representative of the general South Korean child population.

### 2.2. Variables and Measurement

#### 2.2.1. Sociodemographic Variables

Sociodemographic variables included parent and child age, sex, parental education (low, medium, high), household income (below average, average, above average), place of residence, and parental occupational status. Child anthropometric measures (height and weight) were collected by parent report. Parental education was coded as a three-level categorical variable (low, medium, high) for descriptive analyses and as an adjustment covariate in the mediation models. Because the present study was not based on a probability sample, these variables were considered descriptive characteristics and potential covariates rather than sampling weights.

#### 2.2.2. Food Neophobia (FN)

Parent and child food neophobia were assessed using the Food Neophobia Scale (FNS), a widely used measure of reluctance to try or eat unfamiliar foods [3,7,21]. In the present study, the authors translated and culturally adapted the 10-item FNS for Korean use. The procedure included back translation, followed by independent review of the translated versions by the research team, comparison of discrepancies, and reconciliation before the final Korean version was used. The measurement context was informed by cross-national research by Proserpio et al. [22] and by the cross-cultural study of parental and child food neophobia conducted as part of the TITAN Project by Bargiel-Matusiewicz et al. [23]. Responses are recorded on a 5-point Likert scale ranging from 1 (strongly disagree) to 5 (strongly agree). Overall scale scores are computed by averaging item responses, yielding a potential composite score ranging from 1.00 to 5.00, where higher mean values indicate greater severity of neophobic food avoidance. Internal consistency was α = 0.87 for parent FN and α = 0.81 for parent-reported child FN.

#### 2.2.3. Child Eating Behavior Questionnaire (CEBQ)

Child eating behavior was assessed with the Child Eating Behavior Questionnaire (CEBQ), a parent-report instrument using 5-point response categories [11,20,24]. The use of the CEBQ and its scoring framework was informed by prior Korean research, including Park et al. [11], while the present analysis focused on the established Emotional Overeating and Slowness in Eating subscales. Each subscale comprises four items, scored from 1 (never) to 5 (always), giving a theoretical range of 4–20 for each subscale. Item responses outside the valid 1–5 range were treated as missing and were not entered as valid scores. No reverse-coding was applied to either subscale for the present exploratory analysis. Following the item-level scoring audit, the CEBQ Total was defined as the unmodified sum of the two observed subscale scores, yielding a theoretical range of 8–40. Internal consistency was high for both component subscales and the CEBQ Total (Cronbach’s α = 0.88 for Emotional Overeating, 0.86 for Slowness in Eating, and 0.90 for the CEBQ Total). The present analysis therefore uses the established subscales and their unmodified sum as an exploratory composite rather than as a validated global CEBQ score.

#### 2.2.4. Pediatric Symptom Checklist-17 (PSC-17)

Pediatric psychological symptoms were measured using the Korean version of the Pediatric Symptom Checklist-17 (PSC-17), which contains 17 items scored on a 3-point Likert scale: 0 (never), 1 (sometimes), and 2 (often) [25,26,27]. Subscale scores for attention, internalizing, externalizing, and total symptoms are computed by summing item responses for each respective domain, yielding defined ranges: Attention (0–10), Internalizing (0–8), Externalizing (0–10), and Total (0–34) [25,27]. Higher PSC-17 scores indicate increased frequency or severity of behavioral and emotional symptoms. The PSC-17 demonstrated high internal consistency (Cronbach’s α > 0.84).

### 2.3. Statistical Analysis

Descriptive statistics were calculated as frequencies and percentages for categorical variables and means, standard deviations, medians, ranges, skewness, and kurtosis were calculated for continuous variables. Pearson correlations were used for bivariate associations. The prespecified thresholds of |r| ≥0.30 for moderate and |r| ≥0.50 for strong associations were retained. Multivariable linear regression models were specified as follows: Model 0 predicted child FN from parental FN; Model 1 predicted the CEBQ Total from parental FN, child FN, and PSC-17 Total; and Model 2 predicted PSC-17 Total from parental FN, child FN, and the CEBQ Total. Models 1 and 2 were mutually adjusted secondary association models, retained to estimate each child-domain association conditional on the other measured domain; they were not used as the mediation models. Mediation models were separately specified with child age and parental education as covariates. Parental education was coded categorically as low, medium, or high. Continuous predictors were mean-centered where appropriate. The primary association analysis was the parent–child FN association; mediation analyses were considered exploratory. Given the number of correlated tests, the possibility of Type I error inflation was acknowledged and isolated *p*-values were not interpreted as independent confirmation of multiple hypotheses.

Mediation was evaluated using ordinary least-squares path analysis with 5000 bias-corrected bootstrap resamples (using the PROCESS macro version 3.5, Andrew F. Hayes, www.processmacro.org, in IBM SPSS Statistics, version 25.0, IBM Corp., Armonk, NY, USA). Separate mediation models examined the CEBQ Total and PSC-17 Total as outcomes, with child FN as the mediator and child age and parental education as covariates. The indirect association was defined as the product of the parent-FN-to-child-FN and child-FN-to-outcome paths. The primary inferential criterion was the 95% bias-corrected bootstrap confidence interval; an indirect association was considered statistically distinguishable from zero when the interval excluded zero. *p*-values for indirect associations were removed because PROCESS does not provide a bootstrap *p*-value for the indirect effect; the bootstrap confidence interval is therefore the primary inferential criterion. The Sobel test was not used because the sampling distribution of the product of regression coefficients need not be normal.

### 2.4. Ethics

We obtained written informed consent from all parents/legal guardians and written informed assent from all participating children, consistent with the approved study procedures.

## 3. Results

### 3.1. Demographic and Socioeconomic Characteristics

**Figure 1:**
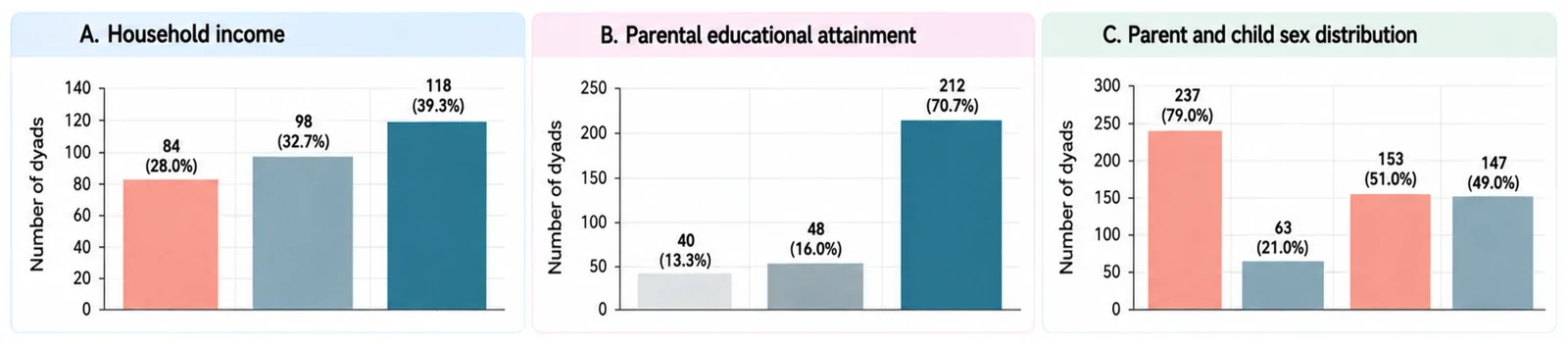
Socio-demographic Profile of the Parent–Child Sample. Note. Panel (**A**) shows household income; Panel (**B**) shows parental educational attainment; Panel (**C**) shows parent and child sex composition. Counts and percentages are taken directly from Table 1. Mean age was 42.02 years (SD = 4.22) for parents and 9.14 years (SD = 1.97) for children.

The final analytical sample comprised 300 parent–child dyads (Table 1 and Figure 1). Caregivers had a mean age of 42.02 years (SD = 4.22), and 79.0% were female. Among parents, 70.7% had high educational attainment, and household income was distributed across below-average (28.0%), average (32.7%), and above-average (39.3%) categories. Children had a mean age of 9.14 years (SD = 1.97), with 49.0% male and 51.0% female. Because participants were recruited through community mental health and counseling centers rather than probability sampling, these characteristics should not be interpreted as population estimates.

### 3.2. Descriptive and Psychometric Performance

**Figure 2:**
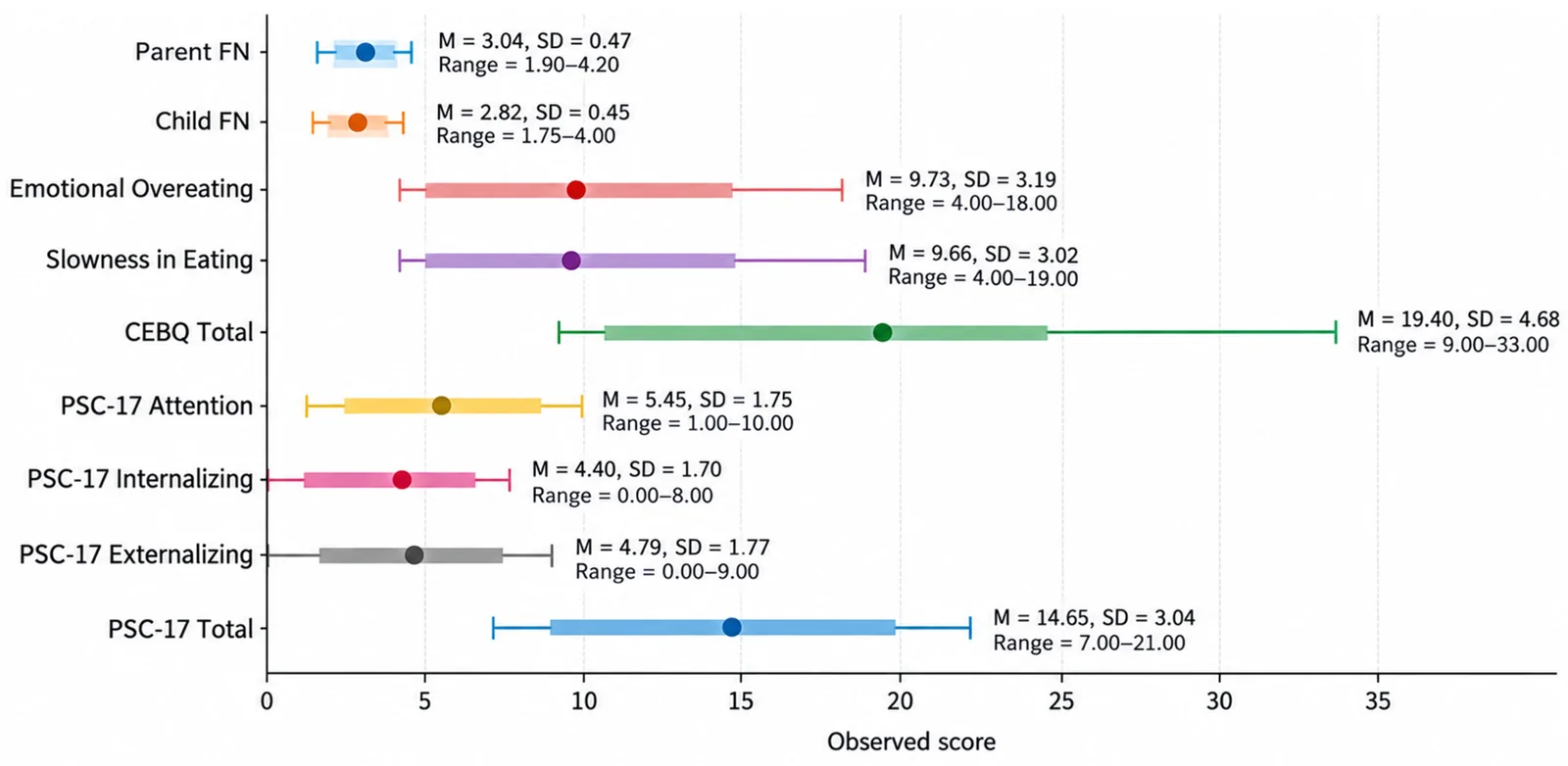
Descriptive Score Profiles After Raw Item-Level Analysis. Note. Mean, standard deviation, and observed minimum–maximum values are displayed for all nine measures reported in Table 2. The CEBQ Total is an exploratory simple sum of Emotional Overeating and Slowness in Eating subscales.

Descriptive statistics from the raw item-level analysis are shown in Table 2 and Figure 2. Parent FN averaged 3.04 (SD = 0.47), and parent-reported child FN averaged 2.82 (SD = 0.45). The Emotional Overeating and Slowness in Eating subscales had means of 9.73 (SD = 3.19) and 9.66 (SD = 3.02), respectively. Their simple sum, the CEBQ Total, averaged 19.40 (SD = 4.68). PSC-17 Total scores averaged 14.65 (SD = 3.04).

### 3.3. Bivariate Associations

**Figure 3:**
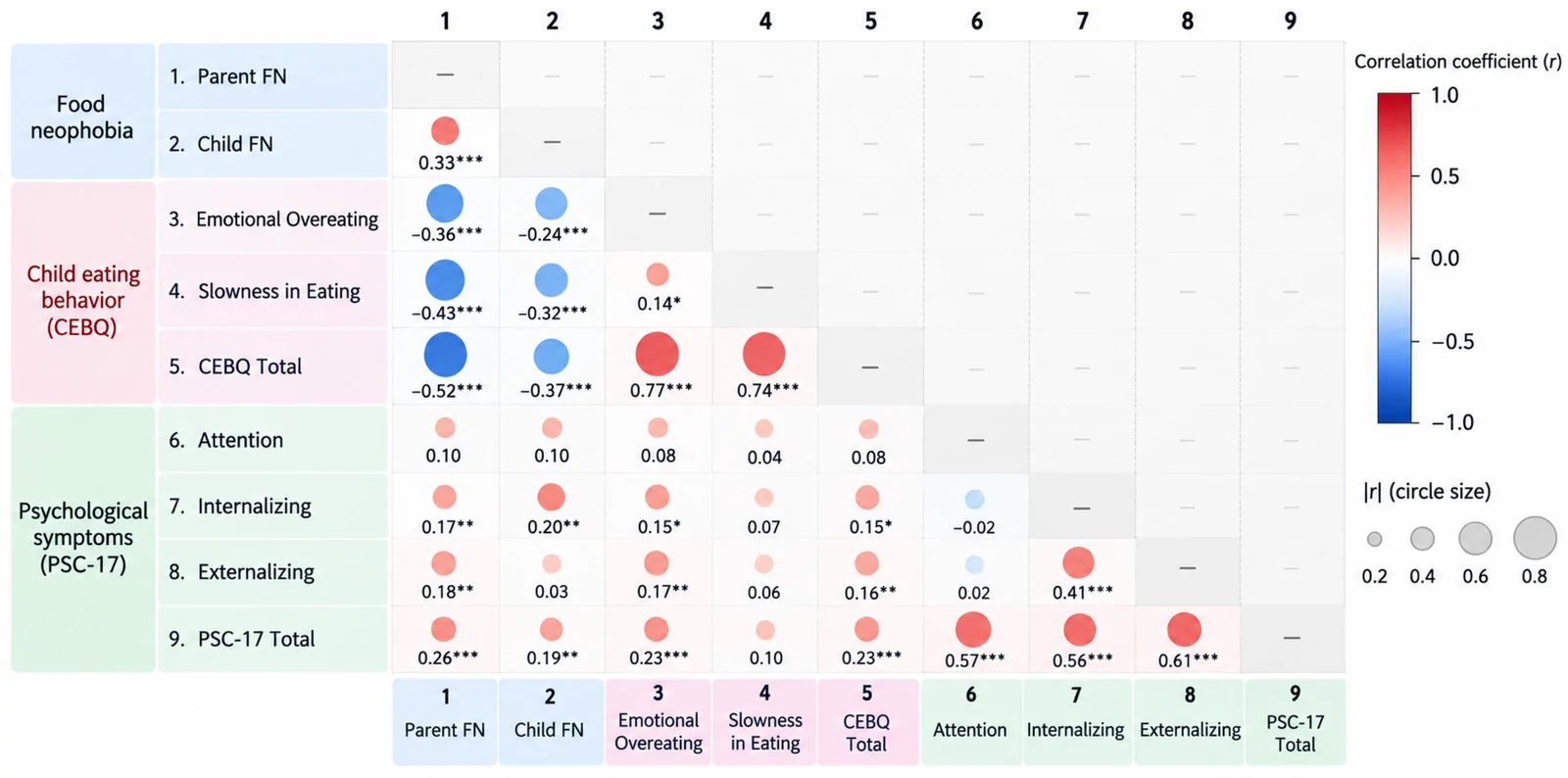
Pearson Correlation Heatmap. Note: The heatmap reproduces the coefficients and significance markers reported in Table 3 exactly. * *p* < 0.05, ** *p* < 0.01, *** *p* < 0.001. CEBQ = Child Eating Behavior Questionnaire; FN = Food Neophobia; PSC-17 = Pediatric Symptom Checklist-17.

Pearson correlations (Table 3; Figure 3) from the analyzed data showed a positive association between parental and parent-reported child FN (r = 0.33, *p* < 0.001), meeting the prespecified threshold for a moderate association. Parent FN was inversely associated with the CEBQ Total (r = −0.52, *p* < 0.001) and positively associated with the PSC-17 Total (r = 0.26, *p* < 0.001). Child FN was inversely associated with the CEBQ Total (r = −0.37, *p* < 0.001) and positively associated with PSC-17 Total (r = 0.19, *p* = 0.001). The two CEBQ subscales were positively correlated (r = 0.14, *p* = 0.018).

### 3.4. Multivariable Regression and Mediation Analysis

Model 0 showed a positive association between parental FN and parent-reported child FN (B = 0.31, SE = 0.05, t = 6.02, *p* < 0.001, 95% CI 0.21–0.42; R^2^ = 0.108). This association is consistent with familial covariation but does not establish temporal ordering or causality. In the secondary mutually adjusted Model 1, higher parental FN (B = −3.83, SE = 0.52, t = −7.33, *p* < 0.001, 95% CI −4.86 to −2.80) and higher child FN (B = −1.84, SE = 0.53, t = −3.48, *p* = 0.001, 95% CI −2.88 to −0.80) were associated with lower scores on the exploratory CEBQ Total after adjustment for PSC-17 Total. PSC-17 Total was positively associated with the CEBQ Total (B = 0.27, SE = 0.07, t = 3.79, *p* < 0.001, 95% CI 0.13–0.41). Model 1 explained 34.7% of the variance (R^2^ = 0.347). Because the CEBQ Total is exploratory, these coefficients should be interpreted as associations rather than effects on a validated global eating-behavior construct.

In the secondary mutually adjusted Model 2, parental FN was positively associated with the PSC-17 Total (B = 1.09, SE = 0.43, t = 2.57, *p* = 0.011, 95% CI 0.26–1.93), whereas the positive association for child FN was marginal and did not reach the conventional 0.05 threshold (B = 0.80, SE = 0.42, t = 1.92, *p* = 0.056, 95% CI −0.02–1.62). The CEBQ Total was not statistically associated with the PSC-17 Total in this model (B = 0.07, SE = 0.04, t = 1.62, *p* = 0.107, 95% CI −0.01 to 0.16). Model 2 explained 9.0% of the variance (R^2^ = 0.090). These mutually adjusted regression estimates are distinct from the covariate-adjusted mediation models and should not be interpreted as causal effects (look at Table 4).

**Table 4:** Multiple Regression Models Predicting Child FN, CEBQ Total, and PSC-17 Total.

Model & Dependent Variable	Predictor	B	SE	t	*p*	95% CI
Model 0: Child FN	Constant	1.87	0.16	11.62	<0.001	1.55, 2.18
	Parent FN	0.31	0.05	6.02	<0.001	0.21, 0.42
	R^2^ = 0.108, Adj R^2^ = 0.105, F (1, 298) = 36.25, *p* < 0.001					
Model 1: CEBQ Total	Constant	32.19	2.57	12.54	<0.001	27.14, 37.24
	Parent FN	−3.83	0.52	−7.33	<0.001	−4.86, −2.80
	Child FN	−1.84	0.53	−3.48	0.001	−2.88, −0.80
	PSC-17 Total	0.27	0.07	3.79	<0.001	0.13, 0.41
	R^2^ = 0.347, Adj R^2^ = 0.341, F (3, 296) = 52.46, *p* < 0.001					
Model 2: PSC-17 Total	Constant	18.74	2.16	8.67	<0.001	14.49, 22.99
	Parent FN	1.09	0.43	2.57	0.011	0.26, 1.93
	Child FN	0.80	0.42	1.92	0.056	−0.02, 1.62
	CEBQ Total	0.07	0.04	1.62	0.107	−0.01, 0.16
	R^2^ = 0.090, Adj R^2^ = 0.080, F (3, 296) = 9.67, *p* < 0.001					

Note. B = unstandardized regression coefficient; SE = standard error; CI = confidence interval. Model 0 predicts child FN from parental FN. Model 1 predicts the CEBQ Total using parental FN, child FN, and PSC-17 Total. Model 2 predicts PSC-17 Total using parental FN, child FN, and the CEBQ Total. Mediation models separately included child age and parental education as covariates. No coefficient is interpreted causally.

### 3.5. Mediation Analysis

The mediation estimates are reported separately from the mutually adjusted regression estimates. The covariate-adjusted PROCESS models and their indirect, direct, and total association estimates are detailed in Table 5. For PSC-17 Total, the indirect association through child food neophobia (FN) was B = 0.212 (SE = 0.118; 95% bias-corrected bootstrap CI, 0.015–0.468). The direct association was B = 1.056 (SE = 0.430, *p* = 0.011; 95% CI, 0.213–1.899), and the total association was B = 1.268 (SE = 0.446; 95% CI, 0.394–2.142). The total point estimate equaled the sum of the direct and indirect point estimates (1.056 + 0.212 = 1.268). For the exploratory CEBQ Total outcome, the indirect association was B = −0.706 (95% bias-corrected bootstrap CI, −1.142 to −0.334), the direct association was B = −4.422 (SE = 0.505; 95% CI, −5.416 to −3.428), and the total association was B = −5.128 (SE = 0.491; 95% CI, −6.093 to −4.162). Both mediation models adjusted for child age and parental education, and indirect-effect confidence intervals were estimated using 5000 bias-corrected bootstrap resamples.

**Figure 4:**
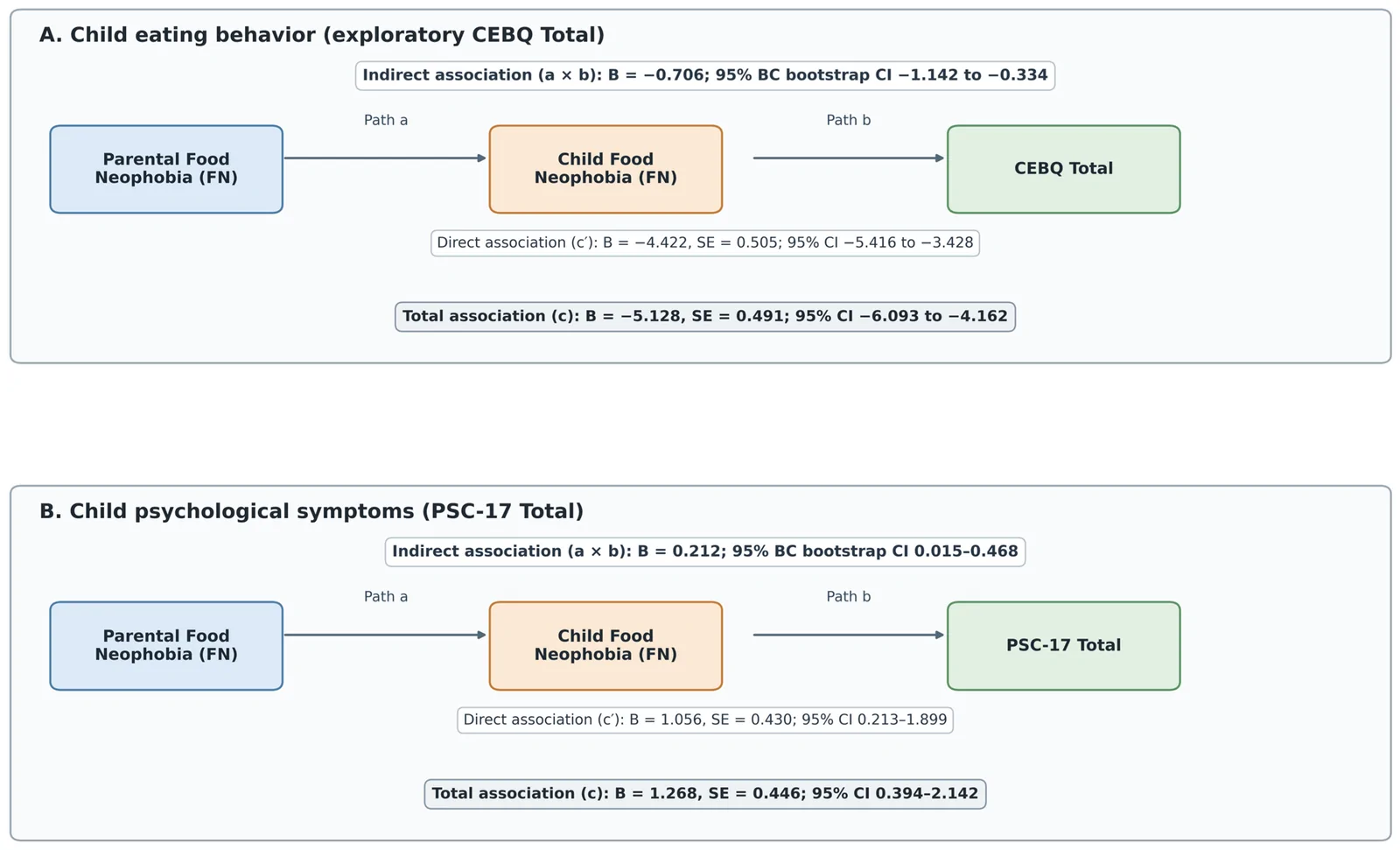
Cross-Sectional Mediation Models for Associations Between Parental FN, Child FN, and Child Outcomes. Note: Figure 4 presents the indirect, direct, and total association estimates. Both models adjust for child age and parental education. These are cross-sectional statistical associations and do not establish causal mediation or intergenerational transmission.

## 4. Discussion

The findings showed a positive association between parental FN and parent-reported child FN in the South Korean parent–child dyads (r = 0.33, *p* < 0.001). Parental FN was also positively associated with PSC-17 Total in the mutually adjusted regression model (B = 1.09, *p* = 0.011), whereas the association for child FN was positive but did not reach the conventional 0.05 threshold (B = 0.80, *p* = 0.056). Bootstrap mediation analyses indicated indirect statistical associations through child FN for both the exploratory CEBQ Total (B = −0.706, 95% CI −1.142 to −0.334) and the PSC-17 Total (B = 0.212, 95% CI 0.015–0.468). These findings are consistent with familial covariation in food-related traits, as reported in studies of genetic and environmental influences on food neophobia and in research examining parent–child food-related characteristics [9,10,16,20,25,27,28]. However, the present cross-sectional data cannot determine whether the observed parent–child association reflects parental modeling, a shared environment, genetic factors, or other familial characteristics.

The broader literature places food neophobia within a spectrum of eating-related behaviors associated with dietary variety, food acceptance, and mealtime experiences. Studies have linked food neophobia with reduced dietary variety and food-related difficulties, although the strength and nature of these associations vary across populations [1,2,7,16,28]. Research from China and Italy also illustrates substantial variation in food-neophobia prevalence and dietary context across settings [8,22]. Food neophobia should nevertheless be distinguished from broader picky or selective eating and from avoidant/restrictive food intake disorder (ARFID), which has specific clinical diagnostic features [4,5,6,18]. Accordingly, the inverse association observed between parental FN, child FN, and the exploratory CEBQ Total should not be interpreted as evidence of a general deficit in child eating behavior; the CEBQ Total is an exploratory composite combining two distinct CEBQ subscales.

The association with psychological symptoms is also relevant but should be interpreted cautiously. Prior work has reported relationships between food neophobia and anxiety-related or self-concept measures, while other research has linked aspects of diet with internalizing and externalizing behaviors in children [19,22]. Broader research on emotional eating and food-related behavior likewise suggests that eating-related characteristics can co-occur with psychological or behavioral factors, although such evidence does not establish a common causal mechanism [11]. In the present study, the PSC-17 findings indicate statistical covariation rather than evidence that food neophobia causes psychological symptoms. The use of PSC-17 Total is supported by established work on screening performance, Korean applications, subscale reliability, and factor structure [25,26,27]. The marginal child-FN b path (*p* = 0.056) and the bootstrap indirect association should be interpreted as different statistical quantities rather than as contradictory findings.

Family and environmental processes provide plausible explanations for the observed associations, but they were not directly tested. Previous studies have examined parental food neophobia together with feeding practices, parental control, and children’s food-related behaviors [16,21]. Research on family food environments has also identified associations with children’s vegetable consumption, while broader integrative work emphasizes the role of family context in children’s feeding difficulties [12,26]. Sensory, behavioral, and family characteristics may further distinguish food neophobia from clinically defined feeding problems [5,6]. In addition, cross-cultural evidence suggests that parent–child food-neophobia associations may be observed across different family and cultural settings [23]. Broader research on family structure and child health-related behaviors provides an indirect reminder that family context can shape child outcomes, although that literature does not directly test food neophobia [18]. Because these mechanisms were not measured comprehensively in the present study, they should be regarded as hypotheses for future investigation rather than explanations established by the current data.

Measurement considerations are important when interpreting the findings. Reviews of food-neophobia instruments have highlighted variation in construct definitions, measurement approaches, and psychometric evidence across studies [3]. Korean research has applied the CEBQ using established subscale-based scoring [11,20], while Korean work on other food-related traits illustrates the importance of evaluating reliability and validity when adapting measures to a specific cultural context [17,20]. Cross-cultural research further suggests that parent–child food-neophobia relationships may be influenced by measurement and contextual differences across countries [23]. In the present study, the established CEBQ subscales were scored according to the prespecified item-response framework, and the CEBQ Total was used as an exploratory composite of the two selected subscales. It should therefore not be interpreted as a validated global measure of adaptive or maladaptive eating behavior.

Several limitations should be considered. First, the cross-sectional design precludes establishing temporal ordering or causal mediation. Second, all child FN and child outcome measures were completed by parents, creating potential common-method and reporting bias; no child self-report of FN was included. Third, recruitment through community mental health and counseling centers may have introduced selection bias and limits generalizability to the general population. Fourth, parental psychopathology, feeding practices, sensory characteristics, and other family-level determinants were not comprehensively measured. Fifth, the CEBQ Total is an exploratory composite based on two established CEBQ subscales. Its scoring follows the established CEBQ item-response and subscale-scoring framework used in prior Korean research [11,20], but the exact Emotional Overeating + Slowness in Eating sum is not a standard global CEBQ score encompassing all validated subscales. The findings involving the CEBQ Total should therefore be interpreted as exploratory and in conjunction with the established component subscales. Sixth, the study does not provide a validated screening cutoff, sensitivity/specificity estimates, or a clinical decision rule for parental FN. Finally, Model 2 explained 9.0% of the variance in PSC-17 Total, indicating that substantial variation remained unexplained by the predictors included in that model. These limitations reduce the extent to which the findings can be generalized or used to infer mechanisms.

The findings therefore provide a basis for hypothesis generation rather than for clinical or intervention recommendations. Future studies should use longitudinal designs to establish temporal ordering, include child self-report and other informants where developmentally appropriate, and measure parental psychopathology, feeding practices, sensory sensitivity, and relevant family-context variables. The established CEBQ subscales should be analyzed separately, and future work should evaluate whether parent–child FN associations remain after accounting for genetic and environmental confounding [3,9,10,24]. Cross-cultural studies may also help determine whether the observed pattern is consistent across populations and whether contextual factors modify these associations [17,20,23].

From an applied perspective, low-pressure exposure to unfamiliar foods and supportive feeding practices are reasonable topics for prospective intervention research, but their effects on FN, eating behavior, or psychological symptoms cannot be inferred from the present cross-sectional data. Existing literature on parental control, feeding difficulties, and ARFID-related presentations underscores the importance of distinguishing general food neophobia from clinically significant feeding problems before considering intervention or screening implications [4,5,6,21,27]. Future intervention studies should therefore use validated outcome measures and prespecified clinical or behavioral criteria, with adequate follow-up to determine whether changes in parental food-related attitudes precede changes in child outcomes.

Overall, the present study adds South Korean parent–child data to a literature that spans dietary behavior, family processes, psychological correlates, and cross-cultural measurement. The observed associations are compatible with a multifactorial model of food neophobia, but the data do not establish intergenerational transmission, causal mediation, or clinical significance. Replication in larger, population-based and longitudinal samples will be important for determining the robustness and temporal ordering of these associations.

## 5. Conclusions

In this South Korean sample of 300 parent–child dyads, parental food neophobia was consistently associated with parent-reported child food neophobia and with both eating-behavior and psychological-symptom indicators. Child food neophobia showed an indirect statistical association linking parental food neophobia with lower exploratory CEBQ Total scores and higher PSC-17 Total scores, suggesting that food-related attitudes may represent a shared family-context marker spanning eating behavior and psychological symptoms. These findings extend the limited Korean evidence by situating food neophobia within a broader parent–child behavioral context rather than treating it solely as an isolated dietary preference. The results also support the value of assessing parental and child food-neophobia patterns together when characterizing children with eating-behavior or psychological concerns and provide a basis for longitudinal studies examining whether family food-related attitudes and experiences precede changes in child outcomes. Because the present data are cross-sectional and child measures were parent-reported, the observed indirect associations should be interpreted as statistical associations rather than evidence of temporal transmission or causality. Future longitudinal research incorporating independent child reports and detailed measures of feeding practices, sensory sensitivity, the family environment, and psychological functioning will be important for clarifying the pathways identified here and determining their clinical and preventive significance.

## Figures and Tables

**Table 1:** Demographic and Socioeconomic Characteristics of Parent–Child Samples.

Variable	Category	*N* (%)/M (SD) *
Parent		
Gender	Male	63 (21.0)
	Female	237 (79.0)
Age (years)		42.02 (4.22)
Size of habitual residence	<10,000	0 (0.0)
	10,000–100,000	20 (6.7)
	100,000–500,000	26 (8.7)
	>500,000	254 (84.7)
Level of education	Low	40 (13.3)
	Medium	48 (16.0)
	High	212 (70.7)
Household income	Below average	84 (28.0)
	Average	98 (32.7)
	Above average	118 (39.3)
Occupation	Housekeeping	84 (28.0)
	Studying	17 (5.7)
	Employment professionally active	134 (44.7)
	Unemployed	20 (6.7)
	Pensioner	12 (4.0)
	Unable to work due to illness or disability	7 (2.3)
	Other option	26 (8.7)
**Child**		
Gender	Male	147 (49.0)
	Female	153 (51.0)
Age (years)		9.14 (1.97)
Height (cm)		134.60 (11.95)
Weight (kg)		32.11 (7.62)

* *N* and % are given for categorical variables; the mean and standard deviation (SD) are shown for continuous variables. Values reflect the full analytical sample unless otherwise specified.

**Table 2:** Descriptive Statistics for Key Psychometric Scales Among Parents and Children.

Measure	Group	M	SD	Median	Min	Max	Skewness	Kurtosis
Food Neophobia	Parent	3.04	0.47	3.10	1.90	4.20	−0.46	−0.42
Food Neophobia	Child	2.82	0.45	2.88	1.75	4.00	−0.04	−0.42
CEBQ Emotional Overeating	Child	9.73	3.19	10.00	4.00	18.00	0.06	−0.01
CEBQ Slowness in Eating	Child	9.66	3.02	10.00	4.00	19.00	0.15	−0.37
CEBQ Total	Child	19.40	4.68	19.00	9.00	33.00	0.32	−0.30
PSC−17 Attention	Child	5.45	1.75	5.00	1.00	10.00	−0.04	−0.34
PSC-17 Internalizing	Child	4.40	1.70	4.00	0.00	8.00	−0.08	−0.58
PSC-17 Externalizing	Child	4.79	1.77	5.00	0.00	9.00	−0.05	−0.47
PSC-17 Total	Child	14.65	3.04	15.00	7.00	21.00	0.16	−0.50

Note. All statistics represent raw values or summed scale indicators unless otherwise indicated. M, mean; SD, standard deviation; Min, observed minimum; Max, observed maximum; Kurtosis, excess kurtosis. CEBQ = Child Eating Behavior Questionnaire; PSC-17 = Pediatric Symptom Checklist-17. Each CEBQ subscale contains four items scored 1–5, giving a theoretical range of 4–20; the exploratory CEBQ Total therefore has a theoretical range of 8–40. The CEBQ Total is an exploratory simple sum of the Emotional Overeating and Slowness in Eating subscales.

**Table 3:** Pearson Correlation Matrix Among Food Neophobia, CEBQ, and PSC-17 Measures.

Variables	1	2	3	4	5	6	7	8	9
1. Parent FN	1.00	0.33 ***	−0.36 ***	−0.43 ***	−0.52 ***	0.10	0.17 **	0.18 **	0.26 ***
2. Child FN	0.33 ***	1.00	−0.24 ***	−0.32 ***	−0.37 ***	0.10	0.20 **	0.03	0.19 **
3. CEBQ Emotional Overeating	−0.36 ***	−0.24 ***	1.00	0.14 *	0.77 ***	0.08	0.15 *	0.17 **	0.23 ***
4. CEBQ Slowness in Eating	−0.43 ***	−0.32 ***	0.14 *	1.00	0.74 ***	0.04	0.07	0.06	0.10
5. CEBQ Total	−0.52 ***	−0.37 ***	0.77 ***	0.74 ***	1.00	0.08	0.15 *	0.16 **	0.23 ***
6. PSC-17 Attention	0.10	0.10	0.08	0.04	0.08	1.00	−0.02	0.02	0.57 ***
7. PSC-17 Internalizing	0.17 **	0.20 **	0.15 *	0.07	0.15 *	−0.02	1.00	0.02	0.56 ***
8. PSC-17 Externalizing	0.18**	0.03	0.17 **	0.06	0.16 **	0.02	0.02	1.00	0.61 ***
9. PSC-17 Total	0.26 ***	0.19 **	0.23 ***	0.10	0.23 ***	0.57 ***	0.56 ***	0.61 ***	1.00

Note. Pearson correlation coefficients (r) are shown in a symmetric matrix using the same row and column order. * *p* < 0.05, ** *p* < 0.01, *** *p* < 0.001. FN = Food Neophobia; CEBQ = Child Eating Behavior Questionnaire; PSC-17 = Pediatric Symptom Checklist-17. The CEBQ Total is an exploratory sum of Emotional Overeating and Slowness in Eating.

**Table 5:** Mediation Analysis: Indirect and Direct Associations of Parental Food Neophobia with Child Outcomes via Child Food Neophobia.

Model & Outcome	Pathway	Effect (B)	SE	95% CI	*p*
CEBQ Total	Indirect Effect (Parent FN → Child FN → CEBQ Total)	−0.706	0.208	[−1.142, −0.334]	—
	Direct Effect (Parent FN → CEBQ Total)	−4.422	0.505	[−5.416, −3.428]	<0.001
	Total Effect (Parent FN → CEBQ Total)	−5.128	0.491	[−6.093, −4.162]	<0.001
PSC-17 Total Score	Indirect Effect (Parent FN → Child FN → PSC-17 Total)	0.212	0.118	[0.015, 0.468]	—
	Direct Effect (Parent FN → PSC-17 Total)	1.056	0.430	[0.213, 1.899]	0.011
	Total Effect (Parent FN → PSC-17 Total)	1.268	0.446	[0.394, 2.142]	—

## Data Availability

The data presented in this study are available on reasonable request from the corresponding author, subject to ethical approval and institutional data governance policies. The data are not publicly available due to privacy and ethical restrictions regarding human subjects.
